# Decreased NK cell immunity in kidney transplant recipients late post-transplant and increased NK-cell immunity in patients with recurrent miscarriage

**DOI:** 10.1371/journal.pone.0186349

**Published:** 2017-10-17

**Authors:** Li Zhu, Mostafa Aly, Haihao Wang, Hristos Karakizlis, Rolf Weimer, Christian Morath, Ruben Jeremias Kuon, Bettina Toth, Gerhard Opelz, Volker Daniel

**Affiliations:** 1 Transplantation-Immunology, Institute of Immunology, University Hospital Heidelberg, Im Neuenheimer Feld 305, Heidelberg, Germany; 2 Department of Hematology, Tongji Hospital, Huazhong University of Science and Technology, Wuhan, China; 3 Nephrology unit, Internal Medicine Department, Assiut University, Âssiut, Egypt; 4 Department of Cardiovascular Surgery, Tongji Hospital, Huazhong University of Science and Technology, Wuhan, China; 5 Department of Internal Medicine, University of Giessen, Klinikstraße 33, Giessen, Germany; 6 Department of Nephrology, University of Heidelberg, Heidelberg, Germany; 7 Department of Obstetrics and Gynecology, University Hospital Heidelberg, Im Neuenheimer Feld 440, Heidelberg, Germany; 8 Department of Gynecological Endocrinology and Reproductive Medicine, Medical University Innsbruck, Anichstraße 35, Innsbruck, Austria; University of Sydney, AUSTRALIA

## Abstract

**Background:**

There is evidence that NK-cell reactivity might affect graft outcome in transplant recipients and pregnancy in women.

**Method:**

NK-cell subsets were determined in whole blood using eight-colour-fluorescence flow cytometry in patients before and after renal transplantation, patients with recurrent miscarriage (RM) and healthy controls (HC).

**Results:**

Patients late post-transplant (late-Tx) with functioning renal transplants showed abnormally low CD56dimCD16+ NK-cells containing both perforin and granzyme (vs HC p = 0.021) whereas RM patients exhibited abnormally high numbers of these cells (vs HC p = 0.043). CD56dimCD16+perforin+granzyme+ NK-cell counts were strikingly different between the two patient groups (p<0.001). In addition, recipients late-Tx showed abnormally low CD8+ NK-cells (vs HC p<0.001) in contrast to RM patients who showed an abnormal increase (vs HC p = 0.008). CD8+ NK-cell counts were strongly different between the two patient groups (p<0.001). Higher perforin+granzyme+CD56dimCD16+ and CD8+ NK-cells were associated with impaired graft function (p = 0.044, p = 0.032). After in-vitro stimulation, CD56dimCD16+ and CD56brightCD16dim/- NK-cells showed strong upregulation of CD107a and IFNy, whereas the content of perforin decreased dramatically as a consequence of perforin release. Recipients late post-Tx showed less in-vitro perforin release (= less cytotoxicity) than HC (p = 0.037) and lower perforin release was associated with good graft function (r = 0.738, p = 0.037). Notably, we observed strong in-vitro perforin release in 2 of 6 investigated RM patients. When circulating IL10+CD56bright NK-cells were analyzed, female recipients late post-Tx (n = 9) showed significantly higher relative and absolute cell numbers than RM patients (p = 0.002 and p = 0.018, respectively); and high relative and absolute IL10+CD56bright NK-cell numbers in transplant recipients were associated with low serum creatinine (p = 0.004 and p = 0.012) and high glomerular filtration rate (p = 0.011 and p = 0.002, respectively). Female recipients late post-Tx exhibited similar absolute but higher relative numbers of IL10+IFNy- NK-cells than RM patients (p>0.05 and p = 0.016, respectively).

**Conclusion:**

NK-cells with lower cytotoxicity and immunoregulatory function might contribute to good long-term graft outcome, whereas circulating NK-cells with normal or even increased cytotoxicity and less immunoregulatory capacity are observed in patients with RM.

## Background

NK-cells represent a heterogeneous population of predominantly cytotoxic effector cells. However, Beilke and Gill reported already in the year 2007 that NK-cells can contribute both to allograft immunity and tolerance [1]. Martinez-Llordella et al. and Li et al. described an increase in transcripts associated with NK cells in the peripheral blood of tolerant liver transplant recipients [2, 3]. Sagoo et al. and Bohne et al. published that tolerant kidney and liver transplant patients displayed an expansion of peripheral blood NK lymphocytes [4, 5]. Kesiraju et al. reported on increased NK- and B-cells, increased serum IL10 and decreased serum interferon-gamma (IFNy) in a kidney transplant patient with operational tolerance [6].

NK-cell increases were also observed in stable long-term kidney transplant recipients [7]. Recently, we reported that renal transplant recipients investigated >1.5 years post-transplant showed higher total NK-cell counts than recipients studied <1.5 years after transplantation [7]. High NK-cells were associated with high glomerular filtration rate and low serum creatinine, and with the occurrence of high numbers of CD4+CD25+CD127-Foxp3+ Treg that co-express the phenotype Helios+IFNy- and appear to have stable Foxp3 expression and originate from the thymus [7]. It follows that high NK-cells late post-transplant are not harmful and might contribute to good graft acceptance. We hypothesized that regulatory NK-cells can be formed late post-transplant and are able to inhibit graft-reactive effector cells. Deniz et al. published in 2008 that regulatory NK-cells are able to suppress antigen-specific T-cell responses [8]. Regulatory NK-cells should be immunosuppressive and less or not cytotoxic, as described for uterine NK-cells [9]. Tissue-resident CD56(bright) NK-cells exhibit low natural cytotoxicity and produce little IFNy upon monokine stimulation [10]. Accumulating evidence indicates that uterine NK (uNK) cells are induced and transformed by sensing signals within their microenvironment to protect the mother from the fetal allograft and support the fetus during its development [11]. Disturbances of this tolerogenic milieu in the uterus and NK-cell alterations are associated with impaired pregnancy, as reviewed by De Carolis et al. [12]. Perricone et al. reported on high levels of NK cells in the peripheral blood of patients with anti-phospholipid syndrome and recurrent spontaneous abortion [13]. NK-cell levels were strongly associated with the week of abortion, showing a trend of earlier onset of abortive events related to higher levels of NK cells [13]. Fukui et al. described that women with recurrent spontaneous abortion and implantation failure showed higher percentages of CD56brightIFNy+TNFα+ NK-cells compared with healthy controls and lower proportions of CD56brightIL4+IL10+ cells, although these NK-cell subsets were very low (<2%) in all groups [14].

Based on our findings and the observations of others we hypothesized opposite effects of NK-cells in transplant recipients and patients with recurrent miscarriage (RM). Long-term transplant recipients with good stable graft function, no current rejection or infection and low immunosuppressive maintenance treatment would be expected to show a reduction of cytotoxic NK-cells and an induction of regulatory NK (NKreg) cells that suppress effector cells by cell contact as well as cytokine release, whereas patients with recurrent miscarriage would be expected to have increased numbers of NK-cells in the blood that are strongly cytotoxic and not or much less immunoregulatory [15].

In the present study, we determined in the blood of RM patients and renal transplant recipients with good and stable long-term graft function CD3-negative NK-cell subsets that co-expressed CD56 and/or CD16 (total NK-cell pool) and produced IFNy, perforin and granzyme as markers of their cytotoxic capability in-vivo, and/or IL10 as an indicator of their potential immunoregulatory role. Moreover, increased expression of the degranulation marker CD107 on the surface of NK-cells and an increase of intracellular IFNy as well as a decrease in the number of perforin+granzyme+CD56dimCD16+ NK-cells during 2h and 6h-stimulation with the tumor cell line K562 served as criterion for the cytotoxic function of NK-cells.

## Methods

### Patients and healthy controls

All patients and controls gave informed consent for the tests performed within this study and the study was approved by the Heidelberg ethical committee (S-225/2014). The study was conducted in adherence to the Declaration of Helsinki. All participants provided their written informed consent to participate in this study. This consent procedure was approved by the local ethics committee. Laboratory staff and healthy blood donors served as healthy controls. Blood from patients was obtained during regular visits in the outpatient clinics of the university hospitals in Heidelberg and Giessen. Demographic data of the patients are summarized in Table 1. Patients received combinations of cyclosporine, tacrolimus, steroids and mycophenolate mofetil as immunosuppressive treatment. RM patients had a history of 3 and more consecutive miscarriages. Female non-pregnant RM patients were routinely screened for (i) anatomical disorders by vaginal ultrasound and hysteroscopy; (ii) endocrinologic dysfunctions [polycystic ovary syndrome according to the Rotterdam criteria, hyperprolactinemia, hyperandrogenemia, insufficiency of the corpus-luteum and thyroidal dysfunctions (hypo- ⁄ hyperthyreosis, thyroid autoantibodies)]; (iii) autoimmunologic disorders (antinuclear antibodies >1:160, anticardiolipin antibodies (IgG and IgM), anti-ß2-glycoprotein IgG and IgM, or lupus anticoagulant); (iv) deficiencies in coagulation factors (protein C, protein S, factor XII, or antithrombin); (v) inherited haemostatic changes (genetic analyses factor V, prothrombin and MTHFR genotype); and (vi) fetal and parental chromosomal disorders (numerical aberrations). Analyses were performed at least 2 months after the last pregnancy. We identified 31 patients with idiopathic RM.

**Table 1:** Demographic data of patients and healthy controls.

	HC (n = 37)	Late post-Tx (n = 27)	Early post-Tx (n = 9)	ESRD (n = 32)	RM (n = 31)
**Ages (years, median, range)**	43 (20–71)	54 (29–69)	58 (43–68)	50 (14–68)	34 (23–41)
**Days post-transplant (median, range)**		1602 (95–2541)	43 (7–78)		
**Sex (n, %)**					
**Female**	21 (57%)	9 (33%)	2 (22%)	14 (44%)	31 (100%)
**Male**	16 (43%)	18 (67%)	7 (78%)	18 (56%)	
**Graft No. (n, %)**					
**First**	-	25 (93%)	8 (89%)	-	-
**Second**	-	2 (7%)	1 (11%)	-	-
**Donor (n, %)**	-			-	-
**Living**	-	19 (70%)	5 (56%)	-	-
**Deceased**	-	8 (30%)	4 (44%)	-	-
**Cold ischemia time (n, %)**	-			-	-
**<3 hours**	-	18 (67%)	5 (56%)	-	-
**≥3 hours**	-	9 (33%)	4 (44%)	-	-
**Serum creatinine (n, %)**					
**<2 mg/dl**	-	21 (78%)	6 (67%)		-
**≥2 mg/dl**	-	6 (22%)	3 (33%)	32 (100%)	-
**GFR (n, %)**					
**<35 ml/min**	-		2 (22%)	32 (100%)	-
**35–50 ml/min**	-	11 (41%)	7 (78%)		-
**≥50 ml/min**	-	16 (59%)			-
**HLA-ABDR mismatch>3 (n, %)**		9 (33%)	3 (33%)		
**CMV IgG+ recipients (n, %)**		11 (41%)	7 (78%)		
**CMV IgG+ donors (n, %)**		14 (52%)	7 (78%)		

Abbreviations: HC, Health Control; Late post-Tx, Late post-transplant recipients; Early post-Tx, Early post-transplant recipients; ESRD, End stage renal disease; RM, recurrent miscarriage.

### Sample collection

Samples were collected from January 2016 to April 2017. 10–20 ml of peripheral blood, anticoagulated with EDTA or heparin, from healthy controls (HC), renal transplant recipients (Tx), end stage renal disease (ESRD) and recurrent miscarriage (RM) patients were collected. 2 ml of peripheral whole blood were used for flow cytometric quantification of lymphocytes subsets, Tregs and NK subsets. From the remaining blood sample, peripheral blood mononuclear cells (PBMC) were isolated using density gradient lymphocyte separating medium-lymphodex (Inno-Train Diagnostic, Germany). PBMC were counted using a hemocytometer, and frozen in a final concentration of 4x10^6^ cells/ml in RPMI medium with 10% dimethylsulfoxide (DMSO) and 20% foetal bovine serum (FBS). Each 2 ml cryogenic tube was filled with 1.5 ml PBMC and transferred into a Nalgene Mr. Frosty Cryo 1°C freezing container (ThermoFisher scientific, Waltham, USA) filled with isopropyl alcohol. The container was placed into a deep-freezer at -80°C for 1–2 days. The cryogenic tubes were transferred into a tank with liquid nitrogen. Frozen cells were used for in-vitro stimulation assays.

### Quantifications of lymphocytes and NK-cell subsets

Blood samples were analyzed immediately after arrival in the lab. CD45+, CD3+, CD4+, CD8+, CD19+, CD16+ and CD56+ lymphocyte subsets were determined as described previously [7]. In the present study additional NK-cell subsets were investigated. 150 μl of whole blood and fluorochrome-labeled monoclonal antibodies against CD45, CD16, CD56, CD3, and HLA-DR were added to each tube whereas CD25, CD69, IL-10R and IgG-isotype controls were added only to certain tubes. All tubes were vortexed briefly and incubated in the dark at room temperature. After an incubation period of 30 min, 2 ml of lysing solution diluted 1:10 (BD Bioscience, Sunnyvale, CA, USA) was added to all tubes. Tubes were vortexed again, incubated in the dark at room temperature for 10 min and the cells were washed twice with 2 ml PBS. Tubes containing only surface markers were resuspended with 300 μl PBS and thereafter ready for measurement. When intracellular proteins were analyzed, cells were permeabilized after staining with surface markers and the lysing process.

To the tubes without Foxp3 monoclonal antibody, 500 μl of BD Perm/Wash buffer II diluted 1:10 (BD Bioscience) was added after the last wash and cells were permeabilized for 10 min. Then, cells were washed with 2 ml PBS and resuspended in 100 μl PBS. Antibody against intracellular determinants such as perforin, granzymeB, IL10, and IFNy, was added. After an incubation period of 30 min, cells were washed twice with PBS, resuspended with 300 μl PBS and then ready for measurement.

With respect to the tubes that will contain Foxp3 monoclonal antibody, 2 ml permeabilization washing buffer (diluted 1:10, Foxp3 Stain Buffer Set, eBioscience) was added and cells were washed after staining with surface antibody and the lysing process. Then, 1000 μl Fixation/ Permeabilization buffer diluted 1:3 (Foxp3 Stain Buffer Set, eBioscience, Darmstadt, Germany) was added. Cells were incubated for 30 min and then washed with 2 ml permeabilization washing buffer. Thereafter, antibody against intracellular determinants such as Foxp3 and IFNy was added. After an incubation period of 30 min, cells were washed twice with 2 ml permeabilization washing buffer. Finally, 300 μl PBS was added to resuspend the pellets.

**Fig 1:**
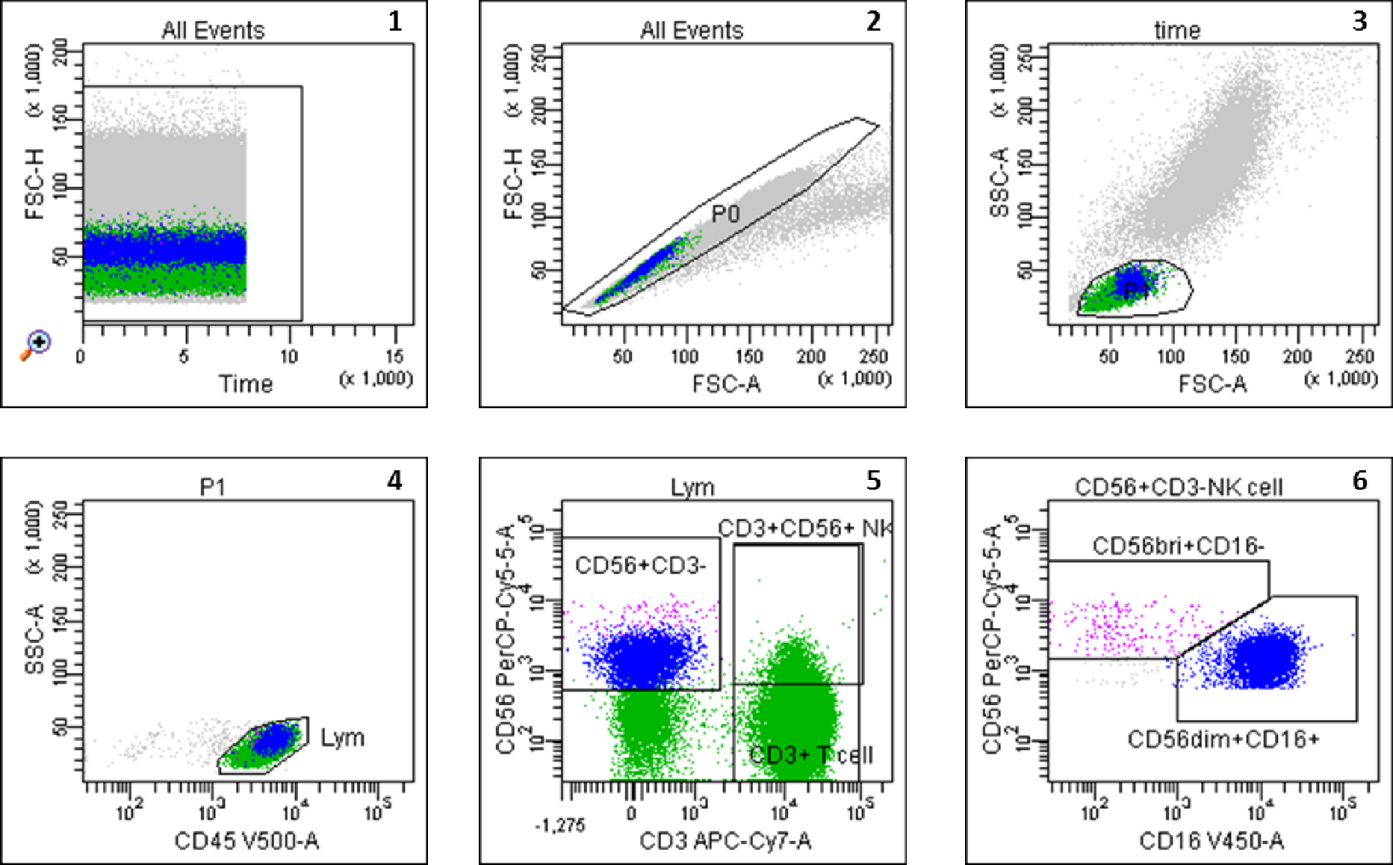
Gating strategy of the two major subsets CD56brightCD16dim/- and CD56dimCD16+ NK-cells. Graph 1 shows the number of analyzed total events in the tube, graph 2 exhibits the exclusion of doublets. PBL were gated according to FSC/SSC (Graph 3) and CD45/SSC dot plot (Graph 4). Only CD3- NK-cells were further analyzed (Graph 5). The 2 major subsets of NK-cells were gated according to the different expression intensity of CD56 and CD16 (CD16/CD56 dot plot, Graph 6). FSC, Forward-scattered light, SSC, Side-scattered light.

Samples were analyzed using eight-color fluorescence and a FACSCanto II triple-laser flow cytometer (BD Biosciences). At least 50,000 lymphocyte events were studied in the initial FSC/SSC dot plot (see gating strategy in Fig 1). Cells were not stimulated for intracellular staining of cytokines. Thus, our data reflect the cytokine production of NK-cells in-vivo.

### Determination of NK-cell function

In order to analyze NK-cell function, stimulation experiments were performed with frozen-thawed PBMC samples from HC, patients early and late post-transplant and patients with ESRD or RM. After collection of 5 to 10 samples, in-vitro assays such as the two-hour degranulation assay and the six-hour multiple response assay were performed. The tumor cell line K562 which lacks MHC class-1 molecules was used to stimulate the isolated lymphocytes for standard NK-cell activity assays [16]. Changes of membrane proteins such as HLA-DR, CD69, CD107a and of intracellular proteins such as perforin, granzyme B, IFNy, and IL10 in NK-cell subsets were measured before and after stimulation.

### Preparation of peripheral blood mononuclear cells and target cells before stimulation

Frozen PBMCs from anticoagulated blood samples were thawed within 1 minute in a 37°C water bath. Pre-warmed 10% RPMI medium was used to dilute the thawed cells. Cells were centrifuged at 300g for 3 min, supernatant was discarded and cell pellets were washed once with 5 ml 10% PRMI. Cells were resuspended in cell culture medium and cell concentration was adjusted to 2×10^6^ cells/ml. Cells were stored overnight in an incubator at 37°C and 5% CO_2_ atmosphere. K562 cell line was incubated at 37°C and 5% CO_2_ and the culture medium was changed 24h before the stimulation experiment.

### Two-hour degranulation assay

PBMC and K562 tumor cell line were spun down and resuspended in 10% RPMI medium. Then, cell numbers were adjusted to 2×10^6^ cells/ml. 150 μl of PBMC suspension were pipetted into each well of a U-bottom 96 well plate. 30 μl of K562 tumor cell suspension were incubated with PBMC at 37°C for 2 hours using an E:T ratio of 5:1. After an incubation period of 1 h, 20 μl of cell culture medium supplemented with Monensin (Golgistop, BD Biosciences) diluted 1:100 were added to each well. After another hour of incubation time, cells were centrifuged at 300 g for 5 min, resuspended in 100 μl of PBS, stained with fluorochrome-labeled monoclonal antibody CD3, CD56, CD16, CD45, CD69, CD107a, CD8 and HLA-DR for 30 min at room temperature in the dark. Thereafter, cells were washed and analyzed using an eight-color fluorescence flow cytometer FACS Canto II (BD Biosciences).

### Six-hour multiple response assay

PBMC and K562 tumor cells were spun down, resuspended in 10% RPMI medium, and adjusted to 2×10^6^ cells/ml. 150 μl of PBMC were pipetted into each well of a U-bottom 96 well plate. 30 μl of K562 tumor cells were added to the PBMC and incubated at 37°C for 6 hours using an E:T ratio of 5:1. After 1 hour incubation time, 20 μl of cell culture medium supplemented with Monensin (Golgistop, BD Bioscience) diluted 1:100 was added. After another incubation period of 5 h, cells were centrifuged at 300 g for 5 min. Cell pellet was resuspended in 100 μl PBS, stained with fluorochrome-labeled monoclonal antibody CD3, CD56, CD16, CD45, and HLA-DR for 30 min at room temperature in the dark. Then, cells were washed and permeabilized using BD Perm/Wash buffer II (BD Bioscience) or Fixation/ Permeabilization buffer diluted 1:3 (Foxp3 Stain Buffer Set, eBioscience, Darmstadt, Germany). After permeabilization, monoclonal antibody against perforin, granzyme B, IL10, IFNy, and Foxp3 was added. Then, samples were incubated for 30 min at room temperature in the dark, washed with either PBS or permeabilization washing buffer and resuspended in 300 μl PBS. Fluorescence of cells was analyzed using an eight-color fluorescence flow cytometer FACS Canto II (BD Biosciences).

## Results

### Lymphocyte subset counts in the blood of dialysis patients, patients early and late post-transplant, RM patients and healthy controls

**Fig 2:**
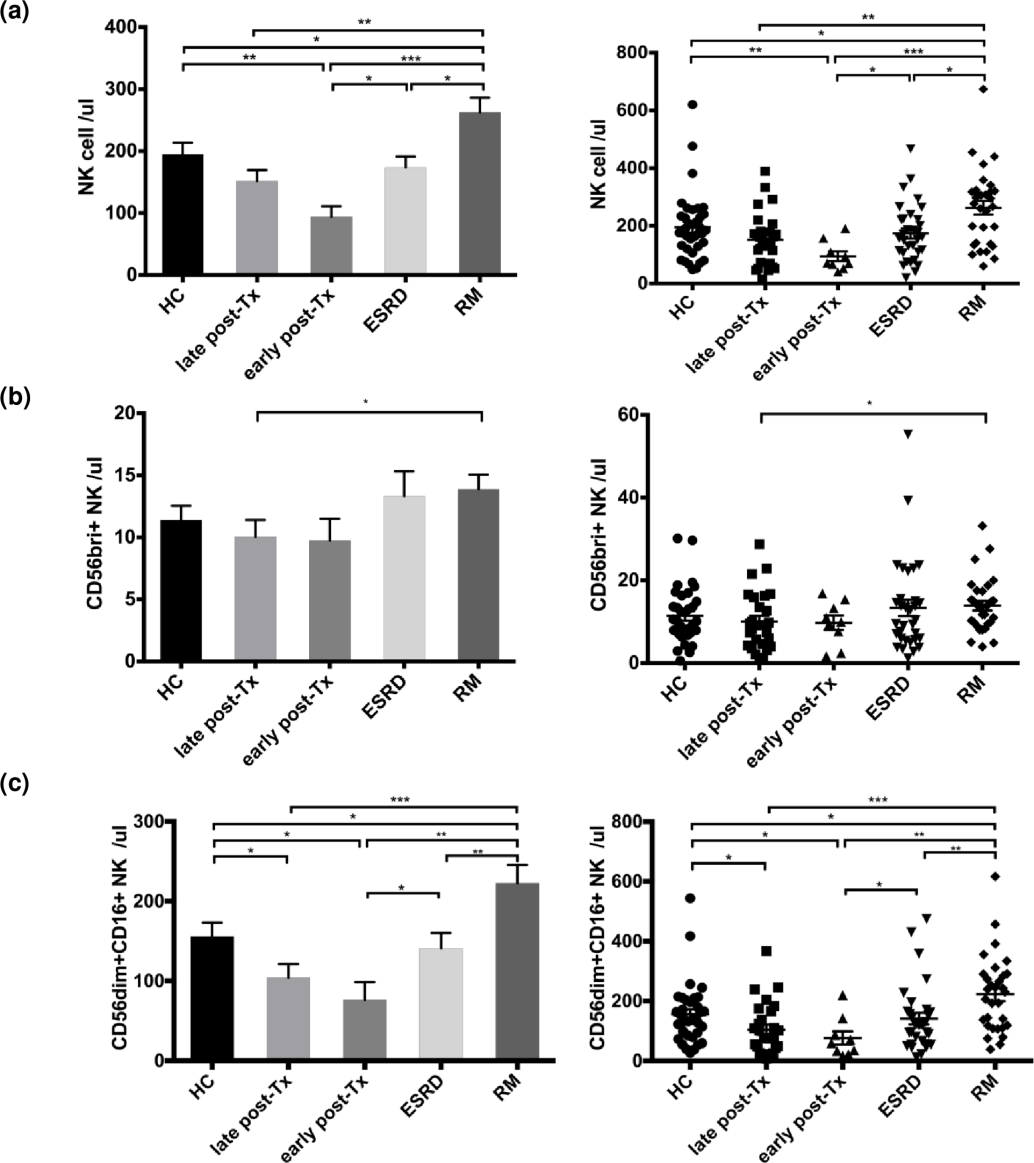
NK-cell subsets in the blood of patients and healthy individuals. Significant differences in absolute cell numbers of (a) CD3- NK-cells co-expressing CD16 and/or CD56, (b) CD56brightCD16dim/- and (c) CD56dimCD16+ NK-cell subsets in healthy controls (HC), renal transplant recipients late post-transplant (late-post-Tx) or early post-transplant (early post-Tx), and in patients with end stage renal disease (ESRD) or recurrent miscarriage (RM). Patients late post-transplant had lower numbers of CD3- NK-cells co-expressing CD16 and/or CD56 (p<0.01), CD56brightCD16dim/- (p<0.05) and CD56dimCD16+ (p<0.001) in the blood than RM patients. *p<0.05, **p<0.01 and ***p<0.001.

Patients late post-transplant had abnormally decreased CD4+ T- and CD19+ B-cell counts (vs healthy controls, all p<0.01) but normal numbers of CD8+ T-lymphocytes and CD3- NK-cells co-expressing CD16 and/or CD56 (vs HC: p>0.05) (Table 2, Fig 2A). In contrast, RM patients showed normal T- and B-lymphocyte counts (vs HC p>0.05) and abnormally increased NK-cell numbers (vs HC p = 0.011). NK-cell counts were higher in RM patients than in kidney graft recipients late post-transplant (p = 0.013) (Table 2, Fig 2A), and even when only the subset of female transplant recipients late post-transplant was compared with RM patients (p = 0.011) (Table 2).

**Table 2:** Absolute counts of lymphocyte subsets in patients and healthy controls.

	HC (n = 37)	Female HC (n = 21)	Male HC (n = 16)	Late post-Tx (n = 27)	Female late post-Tx (n = 9)	Male late post-Tx (n = 18)	RM (n = 31)	ESRD (n = 32)	Early post-Tx (n = 9)	HC vs late post-Tx (p)	HC vs RM (p)	Late post-Tx vs RM (p)	HC vs ESRD (p)	HC vs early post-Tx (p)	ESRD vs late post-Tx (p)	early post-Tx vs late post-Tx (p)	RM vs ESRD (p)	RM vs early post-Tx (p)	ESRD vs early post-Tx (p)	late post-Tx female vs male (p)	HC male vs female (p)	Female HC vs RM (p)	Female late post-Tx vs RM (p)	Female ESRD vs RM (p)
**CD45/μl**	1770± 500	1719± 546	1838± 439	1312± 437	1402± 438	1268± 442	2056± 676	1371± 628	1352± 761	0.000	0.072	0.000	0.002	0.138	0.867	0.985	0.000	0.021	0.801	0.537	0.646	0.061	0.008	0.003
**CD3/μl**	1313± 392	1282± 446	1353± 319	1026± 387	1135± 397	972± 381	1509± 574	1025± 535	1084± 693	0.002	0.125	0.001	0.005	0.414	0.484	0.985	0.000	0.141	1.000	0.328	0.624	0.107	0.058	0.016
**CD4/μl**	816± 233	823± 266	807± 189	638± 281	647± 290	633± 285	974± 410	645± 363	745± 539	0.006	0.128	0.000	0.004	0.570	0.637	0.841	0.000	0.212	0.825	0.918	0.976	0.201	0.022	0.004
**CD8/μl**	452± 216	420± 229	494± 196	369± 149	474± 141	317± 127	488± 198	369± 218	333± 206	0.168	0.346	0.013	0.072	0.154	0.605	0.523	0.013	0.050	0.729	0.012	0.158	0.095	0.961	0.230
**CD16+56/μl**	195± 114	170± 110	228± 115	152± 92	135± 127	160± 72	262± 132	174± 99	94±50	0.070	0.011	0.001	0.406	0.003	0.353	0.096	0.005	0.000	0.017	0.181	0.032	0.005	0.010	0.005
**CD19/μl**	222± 91	227± 89	215± 96	109± 90	109± 71	109± 100	239± 93	125± 110	147± 123	0.000	0.510	0.000	0.000	0.058	0.553	0.432	0.000	0.054	0.637	0.607	0.624	0.648	0.001	0.003
**CD4+CD25+ Foxp3+ Treg /μl**	37±15	39±18	34±11	27±17	26±23	28±15	38±13	38±22	24±17	0.008	0.644	0.006	0.493	0.048	0.040	0.596	0.284	0.043	0.115	0.411	0.560	0.933	0.037	0.082

HC, Healthy Control; Late post-Tx, Late post-transplant; Early post-Tx, Early post-transplant; ESRD, End stage renal disease; RM, recurrent miscarriage. Statistical analysis was performed using Mann-Whitney U test. P-values of <0.05 were marked by yellow and correlations of p<0.01 by orange background.

### CD56dimCD16+ and CD56brightCD16dim/- NK-cell numbers in blood

**Fig 3:**
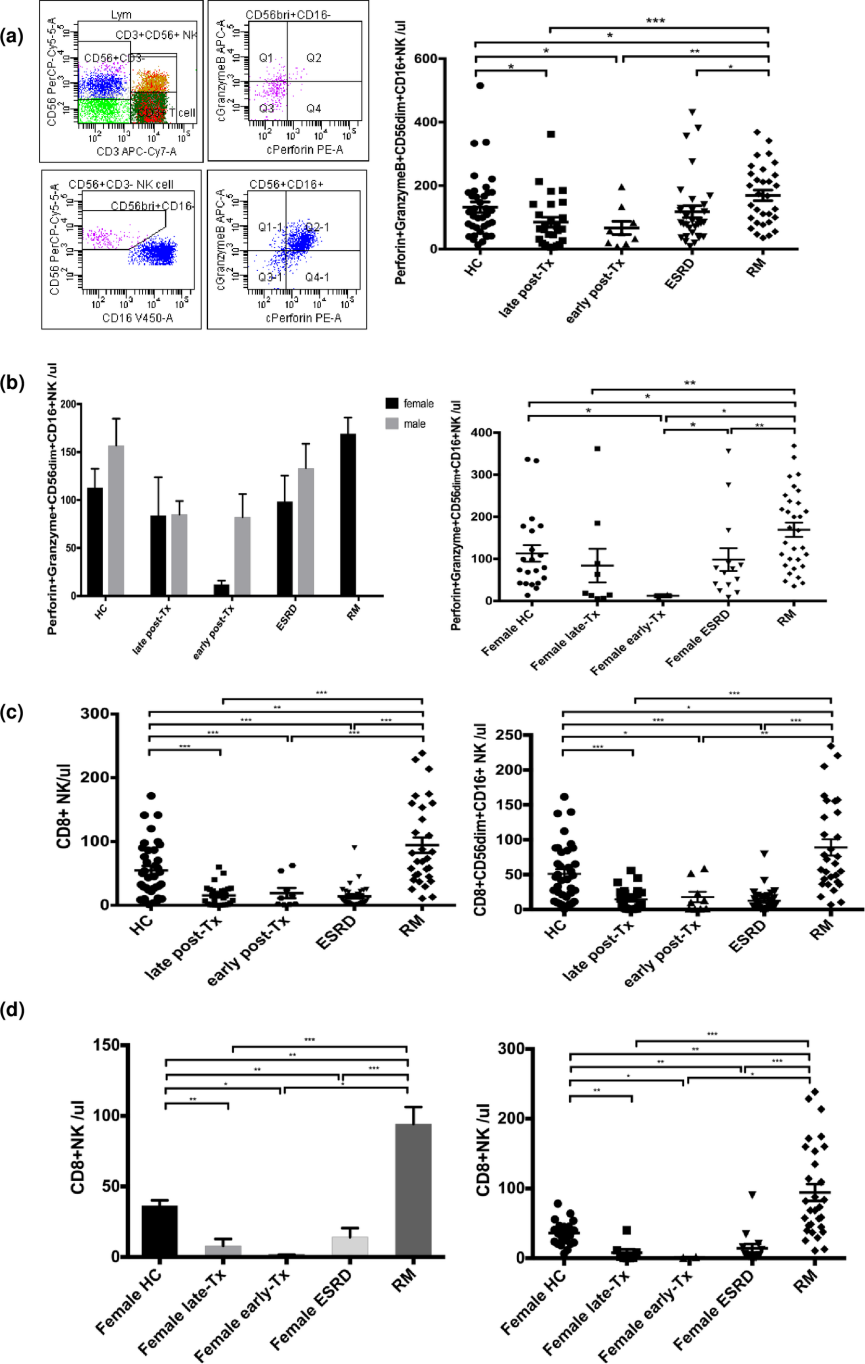
Cytotoxic NK-cell subsets in the blood of patients and healthy individuals, associations with graft outcome. (a) Gating strategy of perforin+granzymeB+CD56dimCD16+ NK-cells and their corresponding absolute numbers in the blood of patients and HC. (b) Absolute numbers of perforin+granzymeB+CD56dimCD16+ NK-cells in the blood of female patients and HC. (c) Absolute numbers of CD8+ and CD8+CD56dimCD16+ NK-cells in the blood of patients and HC and (d) of female patients and HC. Patients late post-transplant and particularly female patients late post-transplant had lower numbers of perforin+granzymeB+CD56dimCD16+ and CD8+ NK-cells in the blood than RM patients (p<0.001).

**Fig 4:**
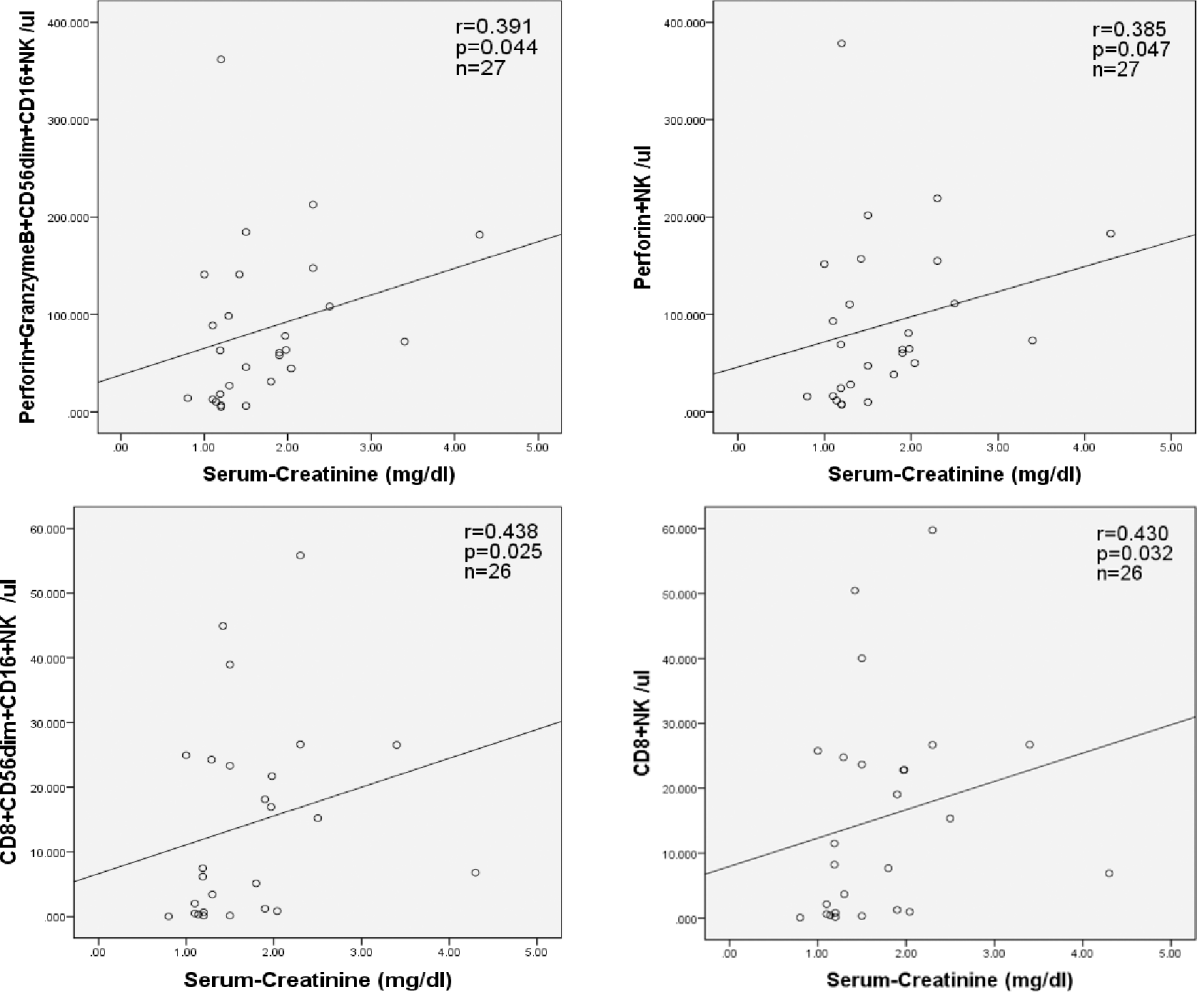
Associations of cytotoxic NK-cell subsets in the blood of patients late post-transplant with graft outcome. Low absolute numbers of perforin+granzymeB+CD56dimCD16+, perforin+, CD8+, and CD8+CD56dimCD16+ NK-cells were associated with good graft function (for all p<0.05).

The pool of NK-cells consists of the major subsets CD56dimCD16+ and CD56brightCD16dim/-. Transplant recipients late post-transplant had significantly lower numbers of CD56dimCD16+ and CD56brightCD16dim/- NK-cells than RM patients (p<0.001 and p = 0.018, respectively) (Fig 2B and 2C). CD56dimCD16+ NK-cells contain more perforin and granzyme than CD56brightCD16dim/- NK-cells and represent the strongly cytotoxic NK subset (Fig 3A). Patients late post-transplant showed abnormally low CD56dimCD16+ NK-cells containing perforin and granzyme (vs HC, p = 0.021) whereas RM patients exhibited abnormally high numbers of this cytotoxic NK-cells subset (vs HC, p = 0.043) and the cell numbers of this particular subset were strikingly different between these two patient groups (p<0.001) (Fig 3A). A significant difference in perforin+granzyme+CD56dimCD16+ NK-cells was also observed when only female transplant recipients (n = 9) were compared with RM patients (n = 31, p = 0.009) (Fig 3B). In addition, transplant recipients late post-transplant showed abnormally low CD8+ NK-cells (vs HC p<0.001), in contrast to RM patients who exhibited an abnormal increase of this particular NK subset (vs HC p = 0.008). CD8+ NK-cell counts were strongly different between the two patient groups (p<0.001) (Fig 3C), and this difference was observed even when only female transplant recipients late post-transplant (n = 9) were compared with RM patients (p<0.001, Fig 3D). Higher numbers of perforin+granzyme+CD56dimCD16+, perforin+, CD8+CD56dimCD16+, and CD8+ NK-cell subsets were associated with worse graft function as determined by serum creatinine levels (for all p<0.05), suggesting an effect of functionally active cytotoxic NK-cells that might affect graft function (Fig 4).

### Cytotoxicity of NK-cells in-vitro

**Fig 5:**
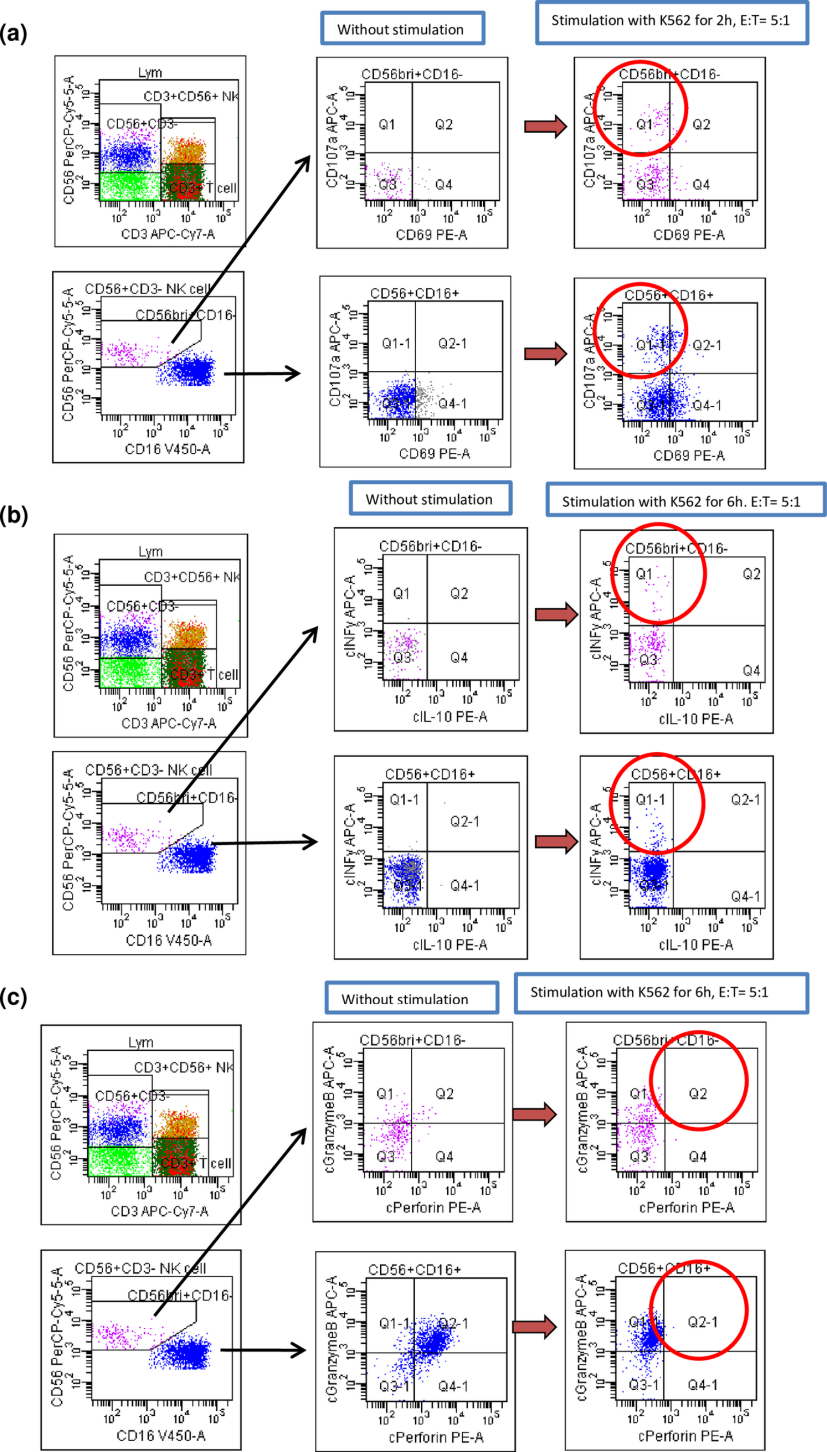
Surface CD107a and intracellular IFNy and perforin after in-vitro stimulation of NK-cells. Gating strategy of the determination of (a) surface CD107a, (b) intracellular IFNy, and (c) intracellular perforin on/in NK-cells after in-vitro stimulation with the tumor cell line K562. CD56brightCD16dim/- NK cells showed stronger upregulation of CD107a during in-vitro stimulation than CD56dimCD16+ NK-cells. IFNy was upregulated and perforin released during stimulation with K562. Granzyme B remained in NK-cells after 6 h in-vitro stimulation.

**Fig 6:**
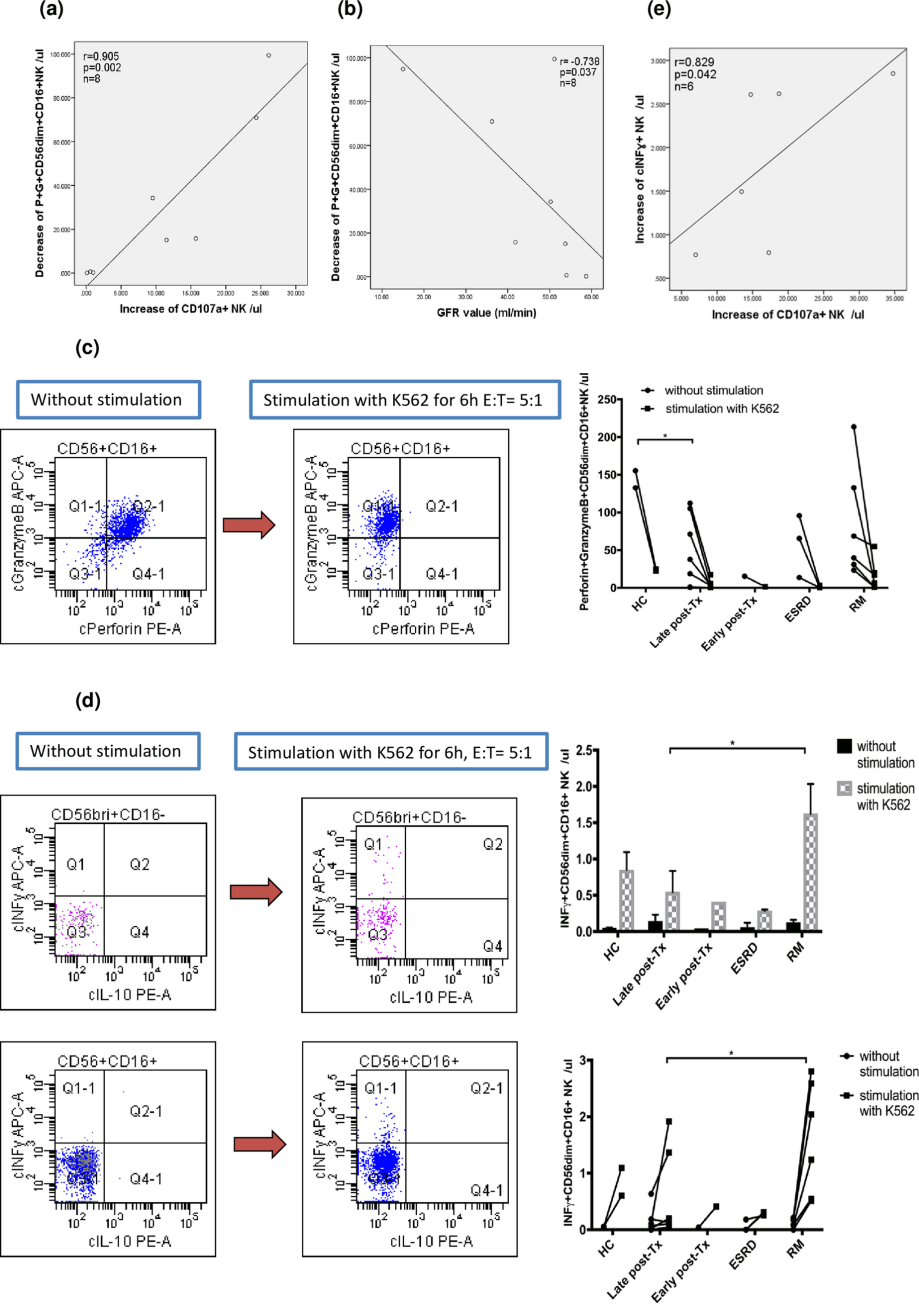
Surface CD107a and intracellular IFNy and perforin after in-vitro stimulation of NK-cells from patients and HC, association with graft outcome. (a) The increase of CD107a on NK-cells was strongly associated with a decrease of perforin+granzyme+CD56dimCD16+ NK-cells (= degranulation and perforin release of NK-cells). (b) The decrease of perforin+granzyme+CD56dimCD16+ NK-cells was associated with high GFR and good graft outcome suggesting an impaired capacity of NK-cells from patients late post-transplant to release perforin (p<0.05). (c) HC showed a stronger perforin release of NK-cells after in-vitro stimulation than graft recipients late-post-transplant (p<0.05). (d) NK-cells of patients late-post-transplant showed a stronger IFNy upregulation during in-vitro stimulation with the tumor cell line K562 than those of RM patients (p<0.05). (e) Low IFNy upregulation during in-vitro stimulation was associated with low upregulation of CD107a on the cell surface of NK-cells obtained from patients late post-transplant (p = 0.042).

In order to study NK-cell cytotoxicity in-vitro, separated PBL were stimulated for 2h and 6h using tumor cell line K562 as a stimulator. Cytotoxic NK-cell subsets in the cell culture were determined. CD56dimCD16+ as well as CD56brightCD16dim/- NK-cells showed strong upregulation of surface CD107a and intracellular IFNy after stimulation (Fig 5A and 5B) whereas the content of perforin decreased dramatically as a consequence of perforin release during cell stimulation (Fig 5C). Release of perforin determined by the reduction of perforin+granzyme+CD56dimCD16+ NK-cell numbers during stimulation with K562 (= decrease of P+G+CD56dimCD16+ NK/μl in Fig 6A) was paralleled by an increase of CD107a+ NK-cells (r = 0.905, p = 0.002) (Fig 6A), indicating conformity in the results of both tests with respect to detection of cell cytotoxicity. Low perforin release was associated with good graft function as determined by the glomerular filtration rate (r = -0.738, p = 0.037) (Fig 6B). When the release of perforin, as determined by a decrease of perforin+granzyme+CD56dimCD16+ NK-cells during in-vitro stimulation with K562 was compared among patient groups and healthy individuals, kidney graft recipients late post-transplant showed less release of perforin than healthy controls (p = 0.037). Notably, we observed very strong perforin release in 2 of the 6 patients with RM (Fig 5C). Moreover, after in-vitro stimulation, patients late post-transplant showed less upregulation of intracellular IFNy than RM patients (p = 0.022) (Fig 6D), substantiating our finding of impaired NK-cell cytotoxicity in patients late post-transplant. Low IFNy upregulation during in-vitro stimulation was associated with low upregulation of CD107a on the cell surface of NK-cells from patients late post-transplant (p = 0.042) (Fig 6E).

In conclusion, patients late post-transplant showed lower numbers of cytotoxic NK-cells in the blood than healthy controls and these NK-cells exhibited impaired cytotoxic function in-vitro. In contrast, patients with RM had abnormally increased numbers of cytotoxic NK-cells in the circulation with normal or even increased cytotoxic function in-vitro.

### Regulatory NK-cells in the blood of patients and healthy controls

**Fig 7:**
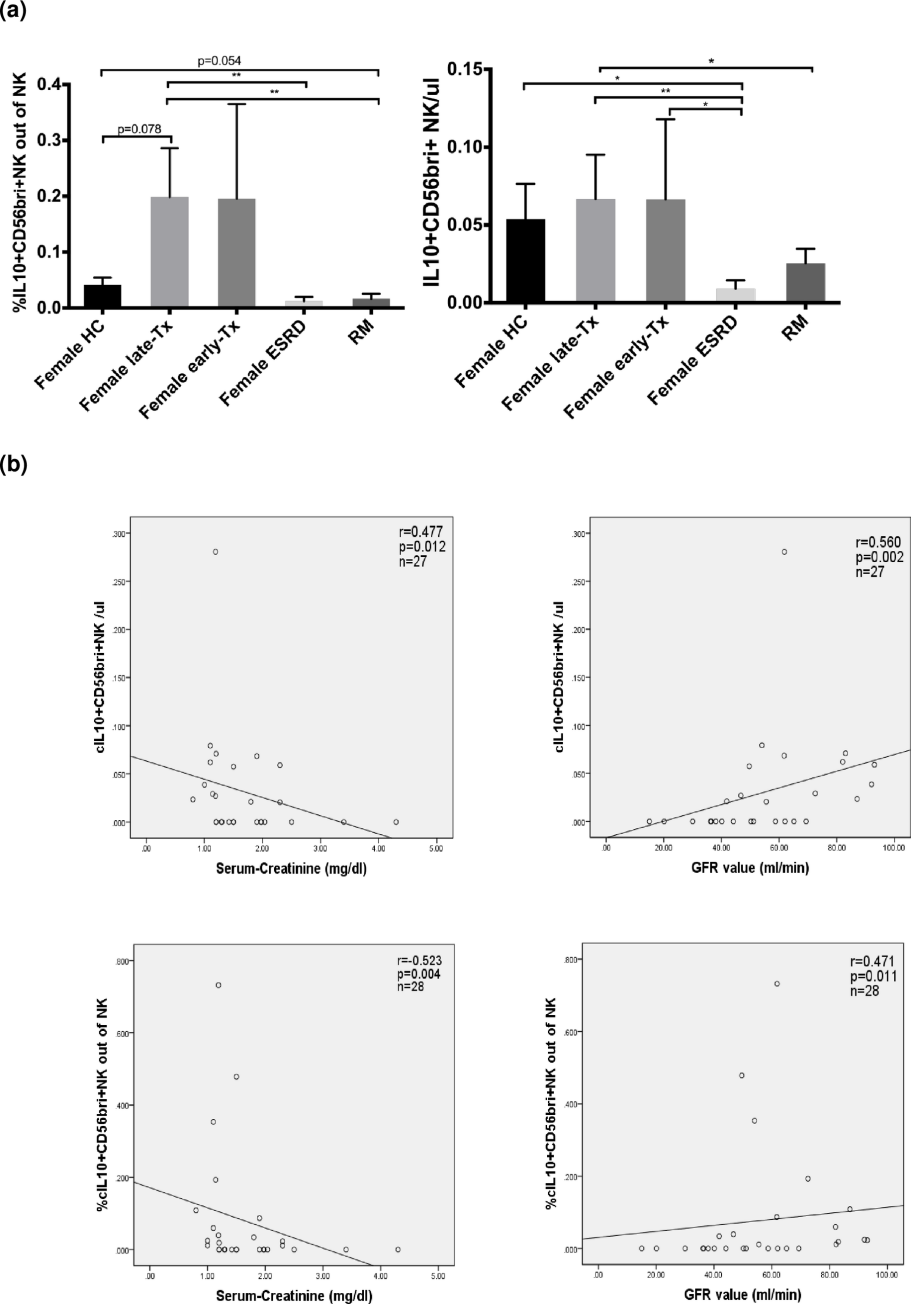
IL10+CD56bright NK-cells in the blood of patients and graft function. (a) Female transplant recipients late post-transplant showed higher relative and absolute counts of IL10+CD56bright NK-cells in the blood than RM patients (p<0.01 and p<0.05, respectively) and (b) relative as well as absolute numbers were associated with low serum creatinine (p = 0.004 and p = 0.012, respectively) as well as high GFR (p = 0.011 and p = 0.002, respectively).

**Fig 8:**
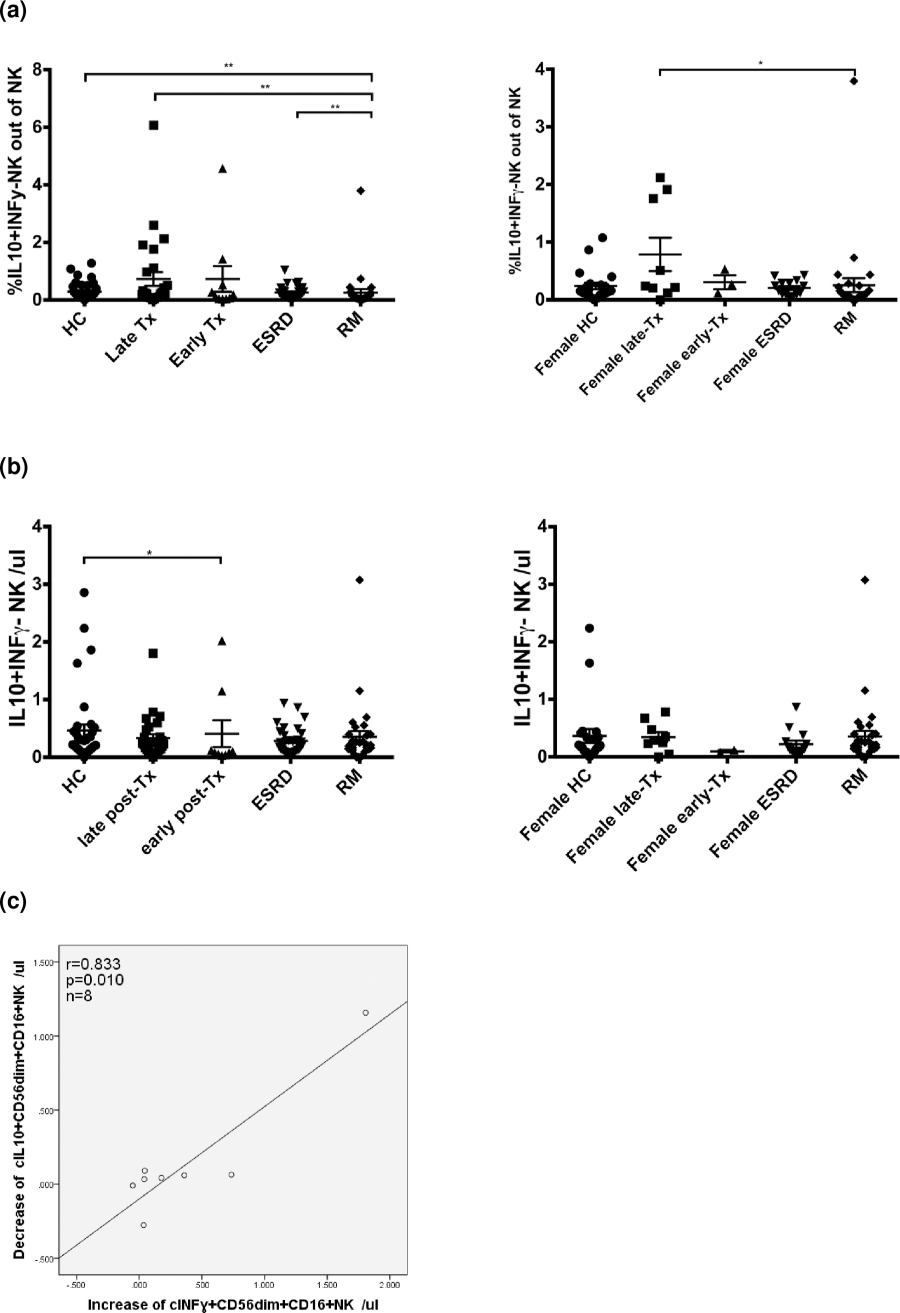
IL10+IFNy-CD56+, IL10+CD56dimCD16+ and IFNy+CD56dimCD16+ NK-cells of patients in-vivo and in-vitro. (a) Relative numbers of IL10+IFNy- NK-cells were significantly higher in patients late post-transplant than RM patients (p = 0.002), even when only female patients were compared (p = 0.016), (b) whereas absolute numbers were similar between the 2 patient groups (p>0.05). (c) When PBL of patients late post-transplant (n = 8) were stimulated with K562 for 2 h, a decrease in absolute numbers of IL10+CD56dimCD16+ NK-cells during stimulation was strongly associated with an increase of IFNy+CD56dimCD16+ NK-cells (r = 0.833; p = 0.01) suggesting that the 2 NK-cells subsets counter-regulate each other.

When IL10+CD56bright NK-cell counts of RM patients were compared with those of female kidney recipients late post-transplant (n = 9), the latter showed significantly higher relative and absolute numbers of this particular cell subset (p = 0.002 and p = 0.018, respectively) (Fig 7A). High relative as well as absolute numbers of IL10+CD56bright NK-cells were associated with low serum creatinine (p = 0.004 and p = 0.012, respectively) and high glomerular filtration rate (p = 0.011 and p = 0.002, respectively) (all Fig 7B) in transplant recipients late post-transplant. When absolute and relative numbers of IL10+IFNy- NK-cells were compared, transplant recipients late post-transplant (n = 27) exhibited similar absolute but higher relative numbers than RM patients (p>0.05 and p = 0.002, respectively), even if only female transplant recipients were compared (n = 9) (p>0.05 and p = 0.016, respectively) (Fig 8A and 8B). The data suggest a trend towards increased regulatory NK-cells in the blood of transplant patients late post-transplant and decreased regulatory NK-cells in the blood of RM patients.

IL-10 and IFNy can be expressed by CD56brightCD16dim/- NK-cells. However, IL-10+ and IFNy+ NK-cells are also detectable in the CD56dimCD16+ NK subpopulation and both subsets appear to counter-regulate each other in-vitro during stimulation with K562. Decreased IL10+CD56dimCD16+ NK-cells were associated with increased IFNy+CD56dimCD16+ NK-cells (r = 0.833; p = 0.010) (Fig 8C).

**Fig 9:**
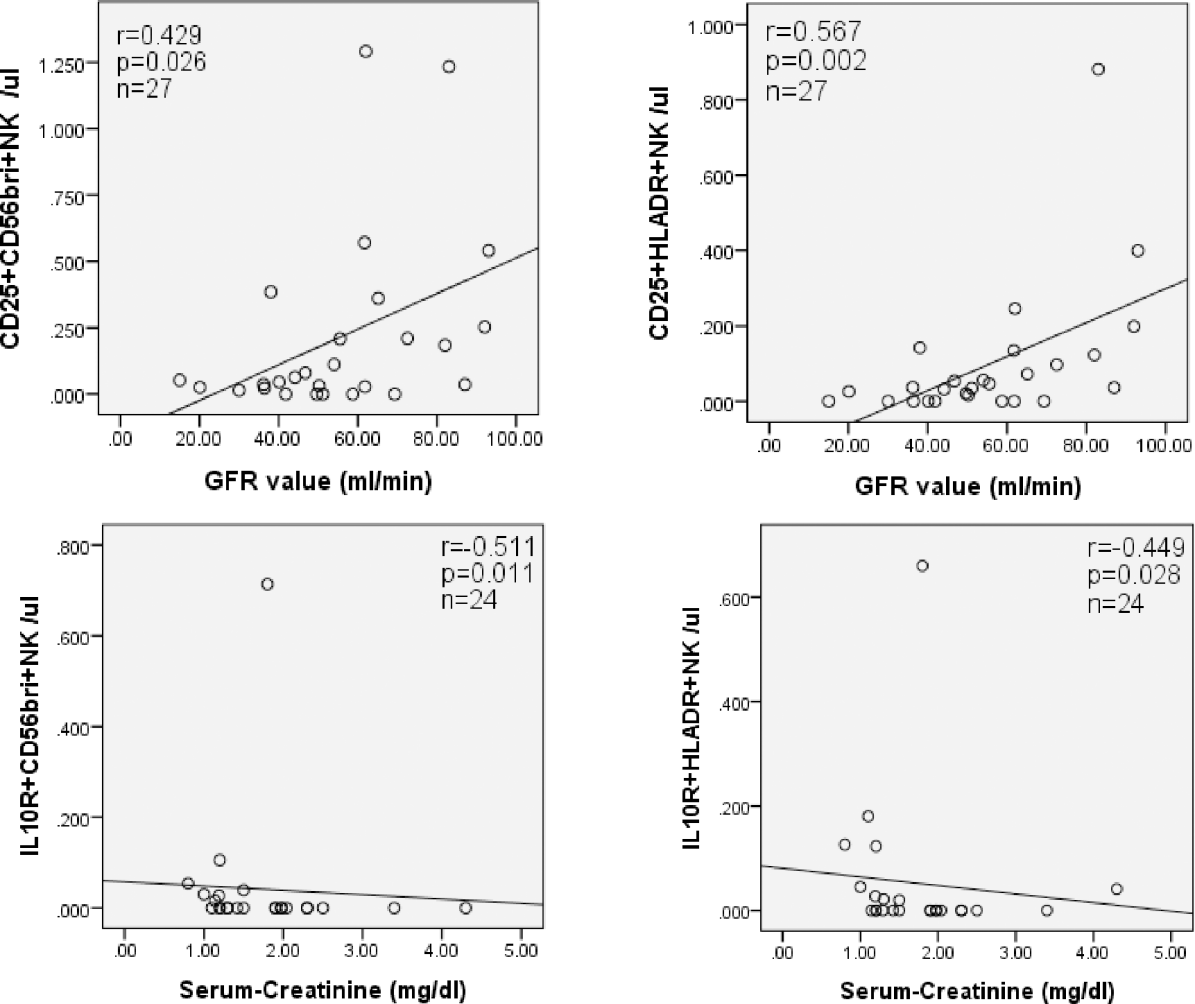
IL10R+CD56bright and CD25+HLADR+ NK-cell subsets in the blood of patients and graft function. High absolute counts of IL-10R+CD56bright and IL10R+HLADR+ NK-cells in graft recipients late post-transplant were associated with low serum creatinine (p = 0.011 and p = 0.028, respectively) whereas high absolute numbers of CD25+CD56bright and CD25+HLADR+ NK-cells in the blood were associated with high GFR (p = 0.012 and p = 0.002, respectively) suggesting an immunoregulatory role of these NK-cells showing an activation phenotype.

In addition, high absolute counts of IL10R+CD56bright and IL10R+HLADR+ NK-cells in graft recipients late post-transplant were associated with low serum creatinine (p = 0.011 and p = 0.028, respectively), and high absolute numbers of circulating CD25+CD56bright and CD25+HLADR+ NK-cells were associated with high GFR (p = 0.012 and p = 0.002, respectively) (Fig 9). We speculate that IL10+CD56bright NK-cells co-express IL10R, CD25 and/or HLADR and exert immunoregulatory function in-vivo. Absolute numbers of IL10R+CD56bright, IL10R+HLADR+, CD25+CD56bright, and CD25+HLADR+ NK-cells were similar in all patient groups and HC (p>0.05) (Table 3).

**Table 3:** Absolute counts of NK subsets in patients and healthy controls.

	HC (n = 34)	Female HC (n = 19)	Male HC (n = 15)	Late post-Tx (n = 27)	Female late post-Tx (n = 9)	Male late Tx(n = 18)	RM (n = 31)	ESRD (n = 32)	Early post-Tx (n = 9)	HC vs late post-Tx (p)	HC vs RM (p)	Late post-Tx vs RM (p)	HC vs ESRD (p)	HC vs early post-Tx (p)	ESRD vs late post-Tx (p)	early post-Tx vs late post-Tx (p)	RM vs ESRD (p)	RM vs early post-Tx (p)	ESRD vs early post-Tx (p)	late post-Tx female vs male (p)	HC male vs female (p)	Female HC vs RM (p)	Female late post-Tx vs RM (p)	Female ESRD vs RM (p)
**CD25+ CD56bright NK /μl**	0.15±0.19	0.10±0.11	0.21±0.25	0.21±0.34	0.37±0.52	0.14±0.19	0.24±0.46	0.16±0.21	0.13±0.31	0.586	0.537	0.449	0.802	0.050	0.599	0.140	0.880	0.033	0.055	0.216	0.171	0.226	0.709	0.573
**CD25+ HLADR+ NK /μl**	0.09±0.13	0.07±0.1	0.12±0.16	0.1±0.18	0.17±0.28	0.06±0.1	0.1±0.24	0.06±0.1	0.06±0.13	0.837	1.000	0.956	0.435	0.135	0.578	0.172	0.549	0.128	0.272	0.081	0.493	0.708	0.215	0.442
**IL10R+ CD56bright NK /μl**	0.10±0.21	0.14±0.26	0.05±0.11	0.04±0.15	0.04±0.04	0.04±0.17	0.13±0.2	0.05±0.11	0.06±0.13	0.150	0.631	0.085	0.902	0.893	0.114	0.334	0.496	0.707	0.811	0.020	0.350	0.930	0.964	0.335
**IL10R+ HLADR+NK /μl**	0.11±0.23	0.12±0.27	0.09±0.18	0.05±0.14	0.08±0.07	0.04±0.15	0.07±0.1	0.04±0.06	0.04±0.08	0.065	0.678	0.203	0.424	0.262	0.295	0.914	0.658	0.505	0.537	0.011	0.526	0.845	0.437	0.427

HC, Healthy Control; Late post-Tx, Late post-transplant; Early post-Tx, Early post-transplant; ESRD, End stage renal disease; RM, recurrent miscarriage. Statistical analysis was performed using Mann-Whitney U test. P-values of <0.05 were marked by yellow background.

### Cytotoxic and immunoregulatory NK-cells and drug doses

**Fig 10:**
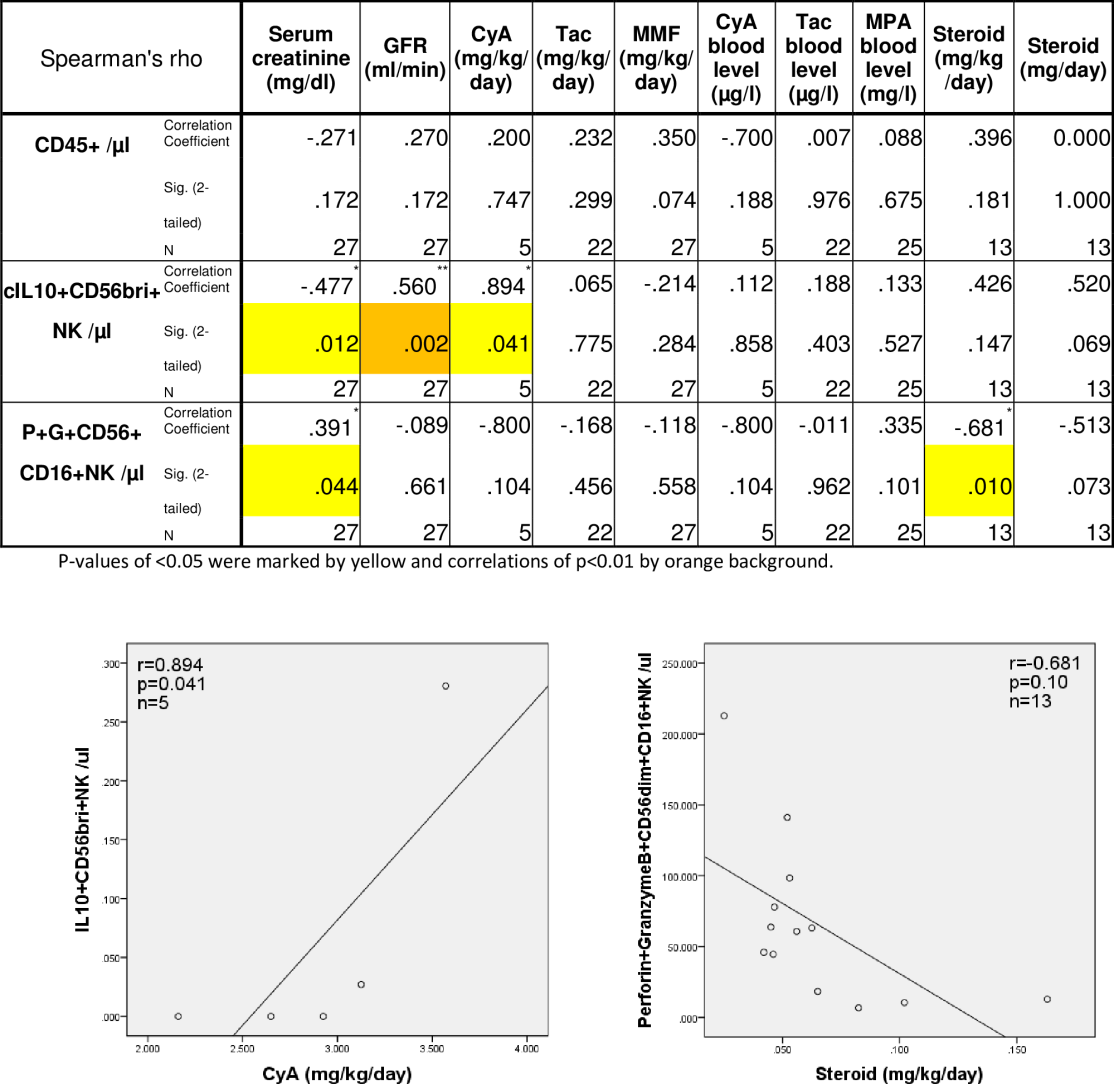
NK-cell subsets and immunosuppressive drug doses. Five patients received cyclosporine and our very preliminary data showed an increase of IL10+CD56bright NK-cells in patients with higher cyclosporine doses late post-transplant (p<0.05). In contrast, 13 patients late post-transplant were treated with steroids and higher steroid doses showed a trend towards lower perforin+granzyme+ CD56dimCD16+ NK-cell numbers in the blood (mg/kg/day p = 0.010; mg/day p = 0.073).

We compared daily doses (with respect to body weight; mg/kg/day) of cyclosporine (n = 5), tacrolimus (n = 22), mycophenolate mofetil (n = 27), steroids (n = 13), steroid doses per day (without respect to body weight; mg/day), and blood trough levels of cyclosporine (n = 5), tacrolimus (n = 22), and mycophenolate mofetil (n = 25) with absolute numbers of total lymphocytes, IL10+CD56bright and perforin+granzyme+ CD56dimCD16+ NK-cells. High IL10+CD56bright NK-cells were associated with high mg/kg/day cyclosporine drug doses (r = 0.894, p = 0.041) and low perforin+granzyme+CD56dimCD16+ NK-cells with high mg/kg/day steroid doses (r = -0.681, p = 0.010) (Fig 10). Low perforin+granzyme+CD56dimCD16+ NK-cell counts were also associated with high mg/day steroid doses (r = -0.513, p = 0.073) (Fig 10). Based on these preliminary data we speculate that higher steroid doses decrease cytotoxic NK-cells in the circulation whereas higher cyclosporine doses might induce potentially immunoregulatory IL10+CD56bright NK-cells.

### Proportions of NK-cell subsets

In the previous paragraphs we compared absolute counts of NK cell subsets. As shown in Table 2, RM patients, patients late post-transplant and HC varied in CD45+ total lymphocyte counts. Therefore, relative numbers of the different NK-cell subsets were analyzed in addition (Table 4). There was no difference in NK-cell proportions among patients late post-transplant, RM patients and HC (p>0.05) or female HC, female patients late post-transplant and RM patients (p>0.05). When we focused on the comparison of female patients late post-transplant with RM patients, RM patients showed lower proportions of CD3+ (p = 0.006) and CD8+ (p = 0.002) T-lymphocytes, higher CD19+ B-lymphocytes (p = 0.014) and similar NK-cells (p>0.05). In addition, RM patients had higher percentages of CD56dimCD16+ (p = 0.034) NK-cells with respect to all lymphocytes. When the proportions of NK-cell subsets within the total NK cell pool were analyzed, RM patients showed higher CD56dimCD16+ (p = 0.011) and lower CD56brightCD16dim/- NK-cells (p = 0.011) than female patients late post-transplant. RM patients also had lower proportions of CD56brightHLADR+ NK-cells (p = 0.005). Interestingly, they had higher percentages of CD8+CD56bright (p = 0.013), CD8+CD56dimCD16+ (p<0.001) and CD8+ (p<0.001) NK-cells. Moreover, they had lower proportions of IL10+CD56bright (p = 0.002), IL10+CD56dimCD16+ (p<0.035), IL10+IFNy- (p = 0.016), IL10+ (p = 0.020), CD25+CD56bright+ (p = 0.019), CD25+HLADR+ (p = 0.004) and CD25+ (p = 0.009) NK-cells than female patients late post-transplant. These data indicate that RM patients possess higher proportions of strongly cytotoxic CD56dimCD16+ and CD8+CD56dim NK-cells whereas potentially immunoregulatory CD56brightCD16- and particularly IL10+, CD25+CD56bright and CD25+HLADR+ NK-cells subsets were lower than in female transplant recipients. To summarize, relative and absolute numbers of the described NK-cell subsets showed similar trends.

**Table 4:** Relative counts of lymphocyte subsets in patients and healthy controls.

	HC (n = 37)	Female HC (n = 21)	late Tx (n = 27)	Female late post-Tx (n = 9)	RM (n = 31)	ESRD (n = 32)	Early post-Tx (n = 9)	HC vs late post-Tx (p)	HC vs RM (p)	Late post-Tx vs RM (p)	HC vs ESRD (p)	HC vs early post-Tx (p)	ESRD vs late post-Tx (p)	early post-Tx vs late post-Tx (p)	RM vs ESRD (p)	RM vs early post-Tx (p)	ESRD vs early post-Tx (p)	Female HC vs RM (p)	Female late post-Tx vs RM (p)	Female ESRD vs RM (p)
**%CD3+ T cells out of lym**	74±5.9	74±6.3	78±9.2	81±8.8	73±6.1	73±12	76±14	0.006	0.339	0.002	0.933	0.244	0.016	0.784	0.448	0.205	0.284	0.501	0.006	0.777
**%CD4+ T cells out of lym**	47±6.8	48±7.0	47±8.7	45±10	47±6.3	46±10	50±13	0.554	0.882	0.444	0.621	0.698	0.451	0.687	0.531	0.697	0.539	0.484	0.770	0.185
**%CD8+ T cells out of lym**	25±7.7	24±7.0	29±9.6	35±10	24±4.6	26±8.9	26±10	0.058	0.608	0.013	0.396	0.967	0.341	0.289	0.193	0.858	0.682	0.793	0.002	0.013
**%CD56+ NK cells out of lym**	11±4.8	10±5.2	12±7.5	10±9.1	13±6.2	14±9.2	10±8.7	0.733	0.145	0.378	0.135	0.318	0.330	0.272	0.788	0.206	0.191	0.058	0.108	0.825
**%CD19+ B cells out of lym**	13±4.0	13±3.6	8.1±6.8	7.9±4.4	12±2.9	8.8±5.2	11±6.5	0.000	0.229	0.000	0.002	0.512	0.192	0.192	0.014	0.820	0.343	0.062	0.014	0.545
**%CD4+CD25+CD127-FOXP3+ Treg out of lym**	2.2±0.85	2.4±1	2.0±1.0	1.7±1.2	1.9±0.42	2.8±1.2	1.7±0.82	0.328	0.208	0.891	0.010	0.150	0.006	0.466	0.000	0.288	0.005	0.033	0.593	0.024
**%CD4+CD25+CD127-FOXP3+ Treg out of CD4+ T cells**	4.3±1.3	4.5±1.6	3.7±1.5	3.3±1.8	3.8±0.9	5.7±1.8	3.3±1.8	0.028	0.114	0.421	0.001	0.017	0.000	0.353	0.000	0.114	0.001	0.121	0.466	0.003
**%CD56bri+NK out of lym**	0.68±0.51	0.79±0.63	0.8±0.59	0.76±0.48	0.71±0.33	1.0±0.7	0.84±0.52	0.643	0.609	0.915	0.023	0.232	0.159	0.619	0.099	0.524	0.392	0.744	0.961	0.377
**%CD56dim+CD16+ NK out of lym**	8.6±4.4	7.5±4.3	8.7±6.9	7.2±8.8	11±5.8	12±9.8	9.2±8.8	0.569	0.052	0.050	0.290	0.406	0.164	0.895	0.441	0.261	0.301	0.009	0.034	0.178
**%CD56bri+CD16- NK out of NK**	8.2±6.9	10±8.4	12.9±12.4	20±17	7.8±5.9	11±7.9	15±14	0.106	0.726	0.095	0.186	0.168	0.859	0.596	0.134	0.163	0.575	0.072	0.011	0.029
**%CD56dim+CD16+ NK out of NK**	92±7.0	90±8.6	87±12	81±17	92±5.9	89±7.9	85±14	0.080	0.868	0.095	0.145	0.145	0.859	0.596	0.134	0.163	0.555	0.129	0.011	0.029
**%CD56bri+HLADR+ NK out of NK**	2.9±1.8	3.3±2.0	4.9±5.3	6.9±6.4	2.1±1.9	2.9±2.7	4.5±4.4	0.397	0.003	0.009	0.186	0.876	0.097	0.921	0.611	0.236	0.315	0.002	0.005	0.447
**%HLADR+ NK out of NK**	11±10	9.0±7.3	12±9.3	13±10	7.9±8.8	9.9±8.8	10±5.6	0.701	0.010	0.013	0.448	0.585	0.286	0.691	0.153	0.114	0.443	0.043	0.067	0.211
**%Perforin+CD56dim+CD16+ NK out of NK**	85±11	83±13	79±14	74±18	83±16	82±12	79±19	0.047	0.790	0.073	0.309	0.346	0.441	0.667	0.483	0.564	0.836	0.678	0.054	0.178
**%Perforin+GranzymeB+CD56dim+CD16+ NK out of NK**	78±14	75±16	75±15	67±18	76±18	77±14	75±18	0.242	0.467	0.534	0.595	0.620	0.486	0.868	0.891	0.976	0.976	0.718	0.102	0.624
**%Perforin-GranzymeB+ NK out of NK**	3.1±3.1	3.0±3.3	7.5±7.2	5.9±5.9	5.1±11	4.8±5.1	6.7±6.1	0.001	0.986	0.002	0.162	0.087	0.062	0.740	0.211	0.122	0.360	0.665	0.067	0.128
**%Perforin+Granzyme+ NK out of NK**	80±14	77±15	77±12	71±14	77±18	79±13	76±19	0.253	0.398	0.564	0.518	0.535	0.505	0.715	0.741	0.976	0.929	0.759	0.124	0.769
**%Perforin+Granzyme-NK out of NK**	7.9±6.7	9.6±7.3	5.1±5.0	8.9±6.7	7.6±5.7	5.8±5.1	4.7±5.4	0.031	0.986	0.039	0.116	0.050	0.594	0.619	0.153	0.042	0.443	0.188	0.593	0.122
**%Perforin+ NK out of NK**	88±9.4	86±11	82±11	80±12	84±16	85±11	81±19	0.020	0.366	0.098	0.200	0.224	0.260	0.765	0.773	0.606	0.723	0.885	0.083	0.339
**%Granzyme+ NK out of NK**	83±12	80±13	84±10	77±12	82±11	83±11	83±15	0.849	0.453	0.362	0.991	0.882	0.813	0.868	0.492	0.485	0.836	0.613	0.237	0.961
**%CD8+CD56bri+ NK out of NK**	3.2±4.3	4.0±4.6	1.5±2.9	2.4±4.8	3.0±2.7	1.5±2.5	1.8±1.5	0.004	0.335	0.000	0.006	0.594	0.714	0.047	0.000	0.142	0.045	0.617	0.013	0.003
**%CD8+CD56dim+CD16+ NK out of NK**	32±16	32±16	13±11	7.2±7.4	38±15	10±11	20±16	0.000	0.152	0.000	0.000	0.054	0.252	0.245	0.000	0.014	0.060	0.201	0.000	0.000
**%CD8+HLADR+ NK out of NK**	2.1±3.4	1.2±1.2	1.2±2.1	1.3±2.8	1.5±1.8	0.7±1.1	1.3±1.6	0.012	0.648	0.036	0.000	0.298	0.456	0.705	0.002	0.303	0.393	0.897	0.053	0.023
**%CD8+NK out of NK**	35±18	36±17	14±12	9.6±11	41±15	12±13	23±16	0.000	0.221	0.000	0.000	0.029	0.215	0.194	0.000	0.010	0.064	0.326	0.000	0.000
**%cIL10+CD56bri+ NK out of NK**	0.04±0.05	0.04±0.06	0.08±0.17	0.2±0.26	0.02±0.05	0.02±0.04	0.12±0.24	0.736	0.018	0.067	0.048	0.523	0.145	0.561	0.677	0.623	0.819	0.054	0.002	0.682
**%cIFNy+CD56bri+ NK out of NK**	0.01±0.02	0.01±0.03	0.13±0.49	0.11±0.22	0.05±0.16	0.01±0.02	0.1±0.18	0.525	0.072	0.331	0.816	0.000	0.411	0.009	0.046	0.028	0.000	0.215	0.985	0.399
**%cIL10+CD56dim+CD16+ NK out of NK**	0.26±0.26	0.22±0.23	0.66±1.2	0.6±0.67	0.26±0.67	0.24±0.19	0.67±1.3	0.431	0.021	0.008	0.686	0.442	0.625	0.296	0.008	0.727	0.262	0.164	0.035	0.091
**%cIFNy+CD56dim+CD16+ NK out of NK**	0.08±0.17	0.06±0.08	0.51±2.1	0.25±0.36	0.12±0.14	0.11±0.14	0.4±0.9	0.690	0.023	0.179	0.110	0.309	0.466	0.615	0.397	0.796	0.824	0.042	0.922	0.990
**%cIL10+cIFNy- NK out of NK**	0.29±0.29	0.24±0.26	0.73±1.3	0.79±0.87	0.25±0.68	0.25±0.21	0.73±1.4	0.357	0.004	0.002	0.963	0.785	0.450	0.407	0.006	0.288	0.637	0.050	0.016	0.062
**%cIL10+ NK out of NK**	0.3±0.29	0.26±0.26	0.74±1.3	0.8±0.89	0.27±0.72	0.26±0.21	0.79±1.4	0.525	0.003	0.003	0.871	0.843	0.486	0.573	0.008	0.316	0.790	0.028	0.020	0.100
**%CD25+CD56bri+ NK out of NK**	0.12±0.15	0.11±0.14	0.47±1.6	1.1±2.7	0.11±0.17	0.16±0.2	0.13±0.29	0.844	0.385	0.308	0.680	0.069	0.923	0.075	0.342	0.119	0.074	0.487	0.019	0.042
**%CD25+CD56dim+CD16+ NK out of NK**	0.03±0.05	0.04±0.07	0.03±0.05	0.04±0.07	0.03±0.07	0.06±0.08	0.01±0.02	0.810	0.687	0.818	0.014	0.618	0.049	0.523	0.047	0.382	0.028	0.495	0.713	0.133
**%CD25+HLADR+ NK out of NK**	0.07±0.09	0.06±0.08	0.15±0.33	0.28±0.48	0.05±0.09	0.05±0.09	0.06±0.12	0.622	0.247	0.153	0.302	0.174	0.196	0.144	0.834	0.304	0.316	0.348	0.004	0.637
**%CD25+ NK out of NK**	0.15±0.17	0.15±0.18	0.5±1.6	1.1±2.7	0.14±0.2	0.22±0.27	0.14±0.3	0.561	0.443	0.202	0.476	0.082	0.976	0.042	0.237	0.100	0.053	0.475	0.009	0.066
**%IL10R+CD56bri+ NK out of NK**	0.09±0.2	0.13±0.25	0.06±0.21	0.04±0.07	0.06±0.1	0.03±0.07	0.06±0.12	0.102	0.955	0.096	0.726	0.947	0.132	0.293	0.546	0.877	0.941	0.539	0.892	0.474
**%IL10R+HLADR+ NK out of NK**	0.09±0.17	0.11±0.2	0.07±0.21	0.11±0.17	0.03±0.05	0.03±0.05	0.03±0.07	0.057	0.375	0.279	0.430	0.197	0.200	0.961	0.843	0.474	0.437	0.416	0.190	0.904

HC, Healthy Control; Late post-Tx, Late post-transplant; Early post-Tx, Early post-transplant; ESRD, End stage renal disease; RM, recurrent miscarriage. Statistical analysis was performed using Mann-Whitney U test. P-values of <0.05 were marked by yellow and correlations of p<0.01 by orange background.

## Discussion

Recently, we showed that renal transplant recipients tested early post-transplant had lower NK-cell counts in the peripheral blood than patients with stable long-term graft function [7]. Late post-transplant, higher NK-cell numbers were associated with better graft function and we hypothesized that NKreg that inhibit effector cells might be induced late post-transplant [7]. Moreover, we hypothesized that NK-cell cytotoxicity decreases late post-transplant. The results of the present study lend further support to these hypotheses. Patients late post-transplant showed lower cytotoxic perforin+granzyme+CD56dimCD16+ NK-cell counts than healthy controls. Patients with higher numbers of perforin+granzyme+CD56dimCD16+ NK-cells and perforin+ NK-cell exhibited worse graft function compared to those with lower numbers, suggesting that these NK-cells are able to harm the graft. Similar results were observed by others who determined NK-cells with a cytotoxicity phenotype in the peripheral blood and in biopsies [17–21]. In our experiments, when NK-cells were stimulated in-vitro using K562, patients with good graft function showed less release of perforin than patients with worse transplant function or healthy individuals. Similar dysfunctional NK-cells were reported in kidney transplant recipients with a diagnosis of cancer [22]. Although kidney transplant recipients with cancer had a higher incidence of acute rejection and cytomegalovirus infection than kidney transplant recipients without cancer, the ability of NK-cells to degranulate CD107a+ cytolytic vesicles was reduced, and the percentage of NK-cells secreting IFNy was decreased [22]. Our data show that patients with good graft function late post-transplant have numerically lower cytotoxic NK-cell counts and these NK-cells are functionally impaired. Although we did not directly measure target cell NK cytotoxicity in the present study, other parameters determined by us, such as increase of the degranulation marker CD107 on the cell surface, release of perforin during in-vitro stimulation, increase of intracellular IFNy, and co-expression of CD8 and CD56bright on the cell surface with simultaneous lack of CD16 expression, are established indicators of an NK-cell subset with strong cytotoxicity [22–26].

IL10 production signals immunoregulatory function of an NK-cell subpopulation [8, 27]. Although the frequency of these so-called NKreg cells is low in the circulation (0.3 cells/μl and 0.7% of all circulating NK-cells in our present study), increased numbers and proportions of these cells were associated with good graft function late post-transplant. Furthermore, the proportion of NKreg was higher in patients late post-transplant than in patients with RM, suggesting an immunoregulatory role of these cells in-vivo. Others observed increased NKreg numbers in patients with breast cancer as compared to healthy individuals and concluded an impaired efficiency of anti-tumor immunity in these patients [28]. In-vitro, IL-10 producing NK-cells were shown to inhibit antigen-specific T-cell responses [8].

Higher frequencies of NKreg cells are present at sites of antigen contact and immune responses as observed in uterus infiltrations during pregnancy, and in spleen, tonsils, and cord blood, and also in patients with autoimmune diseases or cancer [29–35]. Li et al. described the induction of IL10-producing CD56bright NK-cells by administration of an IL-2R blocking antibody in uveitis patients [36]. Our very preliminary data suggest an induction of NKreg cells by the administration of cyclosporine and an inhibition/decrease of cytotoxic NK-cells by treatment with high doses of steroids. We found no indication that calcineurin inhibitors affect perforin and granzyme production of NK-cells and this observation is in agreement with a report of Hoffmann et al. [37]. Although the numbers of individuals in the different patient groups of our study were small, there was a difference in cytotoxic as well as NKreg cells between patients late post-transplant and RM patients as well as to healthy controls. We interpret this as suggesting a “down-regulated” cytotoxic immune system in the first and an “up-regulated” cytotoxic immune system in the latter patient group. Patients early post-transplant showed an even stronger downregulation of NK-cells than patients late post-transplant, a result that we attribute to the higher doses of immunosuppressive drugs administered early post-transplant. ESRD patients prior to transplantation, however, exhibited normal NK subset numbers in the blood.

Because perforin+granzyme+ NK-cells were mainly CD56dimCD16+ whereas IL10+ NK-cells were CD56brightCD16dim/-, these two NK-cell subsets represent separate NK-cell populations. Moreover, perforin+granzyme+ NK-cells released perforin during in-vitro stimulation with K562 whereas granzyme was retained in the cells during 6h stimulation. Cytotoxic function during the first 6 hours of stimulation appears to be based on release of perforin.

## Conclusions

To our knowledge, this is the first report on a comparison of NK-cell subsets with cytotoxic or immunoregulatory phenotype in kidney recipients late post-transplant and patients with RM. The dissimilarity of NK subset numbers and function in these two patient cohorts, and the association of cytotoxic and immunoregulatory NK-cell subsets with late post-transplant graft function, provide evidence for an immunopathological role of cytotoxic and immunoregulatory NK subsets in transplant and RM patients. Further studies need to be done to investigate the interactions of the two NK-cell subsets with each other in-vivo and in-vitro.
